# Propylamin in Rheumatism

**Published:** 1871-06

**Authors:** John M. Gaston


### Miscellaneous.

### Propylamin in Rheumatism.

By John M. Gaston, M. D.

I propose, on this occasion, to simply give you, in a few words, some of the results of my experience in what might be called the specific treatment of acute articular rheumatism.

My reason for bringing this subject before you is, that rheumatism is one of the staple diseases of the country—is almost always present in some form, and in the fall, winter and spring months it often abounds. It is a painful and dangerous disease to have. It has always been a hard thing to manage professionally, baffling, sometimes for weeks, the best directed medical skill—often bringing reproach and discredit upon the profession—and if I can suggest anything that will improve its treatment in your hands, or shorten its duration in the hands of the patient, I shall be repaid.

I need not, on this occasion, enter into any discussion of the pathology of the disease, nor dwell upon its history, its symptoms, diagnosis, nor the ingenious theories of it which, from time immemorial, have entertained the professional mind, interesting though they might be; and I may only refer to the diversity that has always existed among all classes of practitioners with respect to its treatment, to remind you how unsatisfactory a customer it often proves, both to physician and patient, even when best treated, and when neglected or mismanaged, as you have doubtless seen, involving its subject in months of suffering, and important joints and organs of the body in irremediable ruin.

But few statistical observations, that I am aware of, have been recorded with a view to ascertaining the average natural duration of the disease when let alone; but *without* such observations, its reputation as a *durable* and disagreeable disease is pretty well established among us already; and the oft quoted reply of the elder Dr. Warren to a young medical friend, that "the best remedy for rheumatism was six weeks," has, to most minds, forestalled the necessity of such statistical observation, and has often furnished an excuse to calm the conscience of the doctor for his inefficiency, or his inability to cure it, and a melancholy and poor consolation to the patient for his suffering. The truth is, it varies greatly even under apparently the same circumstances, being fickle, and whimsical in a high degree; sometimes beguiling our hopes by an apparent convalescence, only to disappoint and provoke us by renewing its onslought with greater ferocity than ever, perhaps upon the same or other organs, and fortunate will it be for the victim if it do not involve some vital part.

"In 1862 Prof. Flint observed at Belleview Hospital thirteen cases, which were allowed to pursue their course, uninfluenced by therapeutical interference, and the duration of these cases, respectively, from the date of attack to convalescence, was as follows:— In three cases, under fifteen days; in one case, between fifteen and twenty days; in three cases, between twenty and twenty-five days; in three cases, between twenty-five and thirty days; the remaining two cases, forty-five and fifty-six days. The mean duration being a fraction under twenty days."

"Of eighteen cases *treated* in different ways, (but he does not say how,) analysed with reference to duration, in 1856, the minimum duration was seven days, and the maximum was forty-seven days. The mean duration being a fraction over seventeen days."

He also speaks of some cases analysed by him in 1854, in which the mean duration was a fraction over sixteen days.

These data, though very meagre, show that intelligent treatment is of some benefit, although that has been sometimes doubted. A duration of twenty-six days without treatment, is something greater, to say the least, than sixteen or seventeen days with treatment; yet even this is a long time to endure the pains of such a malady. And in passing, I may here allude to some of the more recent and approved modes of treatment in vogue, not wishing, however, to detract from the just merits of any.

Based probably upon correct pathological views, Fuller has introduced the alkaline treatment, which shortens the duration of the disease somewhat, and proportionably lessens the liability to cardiac complication. He deserves great credit. But this treatment involves the necessity of taking such quantities of alkaline substances that I apprehend but few patients in private practice can be induced to undergo the ordeal, even for the relief proimsed.

I have had no experience in the use of the lemon juice treatment. Flint, in his Practice, says this is really an alkaline treatment, but I do not see how it is so. At all events it must be much less disagreeable to the patient than the above.

The treatment of Dr. Davis, of London, by the use of repeated blisters, is favorably spoken of; but under all of these plans I observe the average duration of the disease does not fall much under fourteen days—something of a gain, it is true, yet, if perchance this period might still be shortened, it would be a boon indeed to rheumatic humanity. And it is with this hope that I desire to give you the result of my own experience for several years past in the use of *Propylamin*, an agent not sufficiently known and appreciated, I apprehend, by the profession at large.

It is about eleven years since this article was placed before the profession as a remedy in rheumatism, on the recommendation of Prof. Aryenarious of St. Petersburg, Russia, in a report published in the "Annals of Therapeutics in 1857, p. 74, claiming for it specific powers of a high degree in this disease. He treated with it with success, in two years, between 1854 and 1856, two hundred and fifty cases of rheumatism, acute and chronic, with all sorts of complications, metastatic, pericardial, pleuritic, meningeal, hemphlegic, and paraplegic, and all recovered."

Numerous articles appeared in the journals some years ago confirmatory of these claims for it, and setting forth its uses in other diseases, as neuralgia, etc., but of late years I have not seen much mention made of it in the journals. But my own experience during the past eight years, the time during which I have been using it, has accorded so harmoniously with these reports, and that of the distinguished gentleman named above, as to give me great confidence in its usefulness, and some assurance in recommending it to the profession.

I need not attempt to give you a detailed report of the cases I have treated with it, as that would involve the consumption of too much time, but will, if you please, relate circumstantially only the first case and the last one in which I have used it. And I here take occasion to say that in no single instance has the pain and the soreness of the parts failed to yield completely in 24 or 48 hours, the cure progressing from that time on without interruption—except in two cases, occurring in individuals affected with gonorrhœa at the same time, and even in these two cases it afforded decided relief, but failed further to arrest the disease, and did everything else that I could do, and finally lost sight of both cases. It will be remembered here, that of all forms of rheumatism, gonorrhœal rheumatism is the most inveterate and unamenable to treatment of any form of the disease.

My first experience in the use of this agent occurred in 1863, in the case of an interesting little girl, a child five years of age, in which all the joints of both the upper and lower extremities were successively invaded by the disease, despite my most strenuous efforts to the contrary; and fearing daily the involvement of the heart in the grand ruin, I was in an agony of anxiety and apprehension. I sought counsel, but it availed nothing, as to relieving the case. At last, almost in despair, and scarcely knowing the powers of the remedy for good or evil, and unable to obtain from any source the information I wanted, I brought to bear upon the case, as a sort of forlorn hope, the propylamin, and to my great surprise and gratification, in a little less than forty-eight hours the relief was complete to the aching little limbs, but I regret to say a slight valvular murmer was left in the heart.

I presume every physician, when a case of this disease has gone pleasantly with him, and yielded in apparent obedience to some new agent, has fancied that he has at last found the true remedy for rheumatism, but on the next trial it has perhaps disappointed and deceived him. It had been so with me in former years, and I soon learned to distrust such experience. But in the case of this, the time has been so long, and the success so uniform and so good, that it must be more than a simple coincidence.

My last case occurred a few weeks ago. in the person of John Whitaker, a blacksmith, 30 years of age, involving the feet, knees, wrists, shoulders and elbows successively, with great constitutional disturbance, fever, furred tongue, constipation and loss of appetite. In this case the disease was arrested in a little *over* forty-eight hours—delayed a little beyond the usual time on account of having to stop in the midst of its use, and wait for the administration and operation of a cathartic, the patient being one of those matter of fact individuals, who believe in the importance of the daily performance of that particular function sick or well. His recovery progressed satisfactorily for two or three weeks, but on the very day that he had set to go to work again he suffered a relapse, and became worse than ever. After administering a cathartic, this time in advance to make sure, I put him on the use of the agent, and in forty-eight hours he was all right again.

I may observe here, that my experience with the use of it has been confined to cases of acute rheumatism altogether—and so confident have I become of its powers that I have been in the habit for years, on first diagnosing a case of rheumatism, of promising relief in twenty-four or forty eight hours. The cases have not been so very numerous, but perhaps as many as would naturally come under the attention of a physician in ordinary practice in that space of time—at least one or several a year.

Most cases of acute rheumatism are ushered in by chill, fever, and general disturbance, as well as pain. I usually see that the patient is in a proper condition for the use of the agent, his bowels not constipated. I sometimes order a cathartic, and I frequently premise its use by administering 15 or 20 grains of quinine, in the first twenty-four hours to an adult, after which from 2 to 6 or 8 drops of the liquid propylamin in a tablespoonful of water every two hours for the first twenty-four hours, and at longer intervals the next twenty-four hours, and the cure is accomplished, so far as relief from soreness of the joints and pain is concerned.

The propylamin is found in the shops in two forms, the liquid and the chloride, or muriate. The former is a colorless, transparent liquid, with a singular ammoniacal, and fish-brine odor; is soluble in water, and has an alkaline reaction, and in solution of 2 to 10 drops in a teaspoonful of water is nearly tasteless, and is, so far as I have been able to learn, devoid of poisonous or injurious properties. Its chemical equivalent is $C_6H_9N$.

The chloride is in the form of white crystals, very soluble in water, one grain of which is equivalent in action to about one drop of the liquid.

The agent in either form is somewhat expensive, and that has perhaps been a hindrance to its general use. It formerly sold for five dollars an ounce in this city, but it is cheaper now, costing about three dollars per ounce. I imagine it is sometimes diluted as found in the stores, and if it should fail sometimes on trial, it might be well to bear that in remembrance, and increase the dose.

It is said to exist in cod liver oil, in ergot, in chenopodium, and in sorghum, and is extracted chemically from opium and several other sources, but the most abundant source of its supply is found in herring-brine.

A very convenient formula for its administration is a follows :—

℞ Propylomin . . . . . . 50 to 80 or 100 drops.
Distilled water . . . . . . . . . . . . 8 oz.

M. S.—Dose, tablespoonful every two hours to adult.

This is a larger dose than was used by the authority above referred to, but experience has assured me that it is within bounds of perfect safety.—*Indiana Journal of Medicine.*

